# Simultaneous integrated boost within the lymphatic drainage system in breast cancer: A single center study on toxicity and oncologic outcome

**DOI:** 10.3389/fonc.2023.989466

**Published:** 2023-04-06

**Authors:** Sophie T. Klusen, Antonia Peiler, Georg P. Schmidt, Marion E. Kiechle, Stefan Muench, Rebecca Asadpour, Stephanie E. Combs, Kai J. Borm

**Affiliations:** ^1^ Department of Radiation Oncology, Technical University Munich, Medical School, Klinikum rechts der Isar, Munich, Germany; ^2^ Department of Gynecology and Obstetrics, Technical University Munich, Medical School, Klinikum rechts der Isar, Munich, Germany; ^3^ Deutsches Konsortium für translationale Krebsforschung (DKTK) – Partner Site Munich, Munich, Germany; ^4^ Institute of Radiation Medicine, Helmholtzzentrum München, Munich, Germany

**Keywords:** lymph nodes, breast cancer, simultaneous integrated boost, toxicity, axillary therapy, nodal positivity

## Abstract

**Background and purpose:**

In breast cancer patients, the increasing de-escalation of axillary surgery and the improving resolution of diagnostic imaging results in a more frequent detection of residual, radiographically suspect lymph nodes (sLN) after surgery. If resection of the remaining suspect lymph nodes is not feasible, a simultaneous boost to the lymph node metastases (LN-SIB) can be applied. However, literature lacks data regarding the outcome and safety of this technique.

**Materials and methods:**

We included 48 patients with breast cancer and sLN in this retrospective study. All patients received a LN-SIB. The median dose to the breast or chest wall and the lymph node system was 50.4 Gy in 28 fractions. The median dose of the LN-SIB was 58.8 Gy / 2.1 Gy (56-63 Gy / 2-2.25 Gy). The brachial plexus was contoured in every case and the dose within the plexus PRV (+0.3-0.5mm) was limited to an EQD2 of 59 Gy. All patients received structured radiooncological and gynecological follow-up by clinically experienced physicians. Radiooncological follow-ups were at baseline, 6 weeks, 3 months, 6 months and subsequent annually after irradiation.

**Results:**

The median follow-up time was 557 days and ranged from 41 to 3373 days. Overall, 28 patients developed I°, 18 patients II° and 2 patients III° acute toxicity. There were no severe late side effects (≥ III°) observed during the follow-up period. The most frequent chronic side effect was fatigue. One patient (2.1 %) developed pain and mild paresthesia in the ipsilateral arm after radiotherapy. After a follow-up of 557 days (41 to 3373 days), in 8 patients a recurrence was observed (16.7%). In 4 patients the recurrence involved the regional lymph node system. Hence, local control in the lymph node drainage system after a median follow-up of 557 days was 91.6 %.

**Conclusion:**

If surgical re-dissection of residual lymph nodes is not feasible or refused by the patient, LN-SIB-irradiation can be considered as a potential treatment option. However, patients need to be informed about a higher risk of regional recurrence compared to surgery and an additional risk of acute and late toxicity compared to adjuvant radiotherapy without regional dose escalation.

## Introduction

A positive lymph node status in breast cancer is associated with a significant decrease of the 5 year-survival-rate (1). Nevertheless, in lymph-node positive patients, locoregional treatment of the lymph node drainage system has the potential to decrease the rate of distant and local recurrences and improve the oncological outcome (2, 3). In recent years several large trials have investigated the optimal role of radiotherapy and axillary surgery in this setting:

The AMAROS trial showed comparable local control rates for breast cancer patients with positive sentinel lymph nodes after axillary lymph node dissection (ALND) or regional nodal irradiation (RNI) (4). Further, the results of the Z0011 trial suggest that even axillary incidental lymph node irradiation during tangential whole-breast-irradiation may suffice to achieve similar oncologic outcomes after positive “sentinel lymph node biopsy (SLNB) only” compared to ALND (5, 6). The major advantage of SLNB (+/- radiotherapy) over ALND is the reduction of side effects such as lymphedema and shoulder mobility dysfunction, due to a less invasive approach (7). Thus, in patients with clinically node-negative disease (cN0) without neoadjuvant chemotherapy, “SLNB only” (+/- RNI) has become the standard therapy.

For patients with clinically involved lymph nodes (icN+) however, axillary dissection remains the preferred therapy according to current guidelines. ALND usually comprises resection of level I and II of the axilla. Due to the questionable oncological benefit of an extensive lymphadenectomy (> 10 LN) and the associated toxicity, the trend is towards more limited lymph node removal in ALND (8, 9). A potential alternative to ALND in clinically nodal positive patients is the concept of targeted axillary dissection (TAD). Here, biopsy confirmed axillary nodes are being clip-marked prior to surgery and removed in addition to the sentinel lymph nodes. This concept is primarily being used after neoadjuvant chemotherapy. Recent studies have shown promising results using this approach in patients with clinical positive lymph nodes (10–12).

The progressive de-escalation of axillary surgical therapy and improved resolution of diagnostic imaging prior to radiotherapy lead more frequently to a detection of morphologically abnormal lymph nodes remaining within the axilla levels I-IV and the internal mammary region after completion of surgical treatment. In case of confirming a suspect finding (e.g. in ultrasonic examination), further procedure should be discussed in an interdisciplinary tumor board.

In some cases, remaining pathological lymph nodes or areas at risk cannot be addressed surgically, e.g. due to previously repeated interventions or refusal by the patient. According to the NCCN guidelines, in these cases, a supplemental boost to gross residual lymph nodes can be delivered (13). However, evidence supporting this recommendation is lacking. Even though large randomized trials (e.g. MA.20 trial and the EORTC 22922), have shown that regional lymph node radiotherapy is improving locoregional control rates after surgery, none of these randomized trials have investigated a dose escalation for clinically apparent residual lymph node metastases.

Compared to a sequential boost, the use of simultaneous integrated boost (SIB) allows to keep the total duration of the treatment the same and enables a better homogeneity in dose distribution (14). The use of SIB for pathological lymph nodes is well established in radiooncological treatment concepts of other cancer entities such as prostate carcinomas, head and neck cancers and cervical cancer (15–17). Nevertheless, to our knowledge, no previous data on SIB-irradiation on the lymphatic system is available for breast cancer patients.

Thus, in this study, we aim to provide preliminary data on oncological outcome and toxicity after the use of SIB irradiation (LN-SIB) of radiographically suspect lymph nodes (sLN) in breast cancer patients.

## Materials and methods

### Patient collective

We included 48 adult women (n=47) and men (n=1) with histologically confirmed invasive breast cancer and residual clinical positive lymph nodes after surgery in this retrospective study. The patients gave their informed consent for treatment and the study was approved by the local ethics board (2022-364-S-NP).

The mean patient age at time of diagnosis was 55 years and ranged from 30 to 83 years. Initial tumor stages were predominantly stadium II (n=19) and III (n=23). Beyond, we included 4 patients with affection of cervical lymph nodes (stadium IV). Most patients received either mastectomy (60,4 %, n=29) or breast-conserving surgery (BCS, 31.25 %, n=15) as primary treatment of the breast. In 30 (62.5 %) patients, a neoadjuvant systemic therapy was applied. A concomitant systemic therapy was performed in 14.6% of patients (n=2 Capecitabine, n=1 Olaparib, n=3 endocrine therapy, n=1 combined chemotherapy/antibody therapy (Trastuzumab and Capecitabine)).

Primary treatment of the axillary lymph nodes had been ALND in 30 patients (62.5 %), SLNB in 13 patients (27.1 %) and TAD in one patient (2 %). Median number of resected lymph nodes were 12, (range 1-33), with a median of 2 positive lymph nodes (range 0-25). 4 patients (8.3 %) did not receive any specified axillary treatment prior to radiotherapy due to metastatic breast cancer. In all patients, residual sLN were clinically detected prior to radiotherapy (in planning CT, MRI or PET-CT). In 44 cases, postoperatively detected LNs showed radiographically pathological features. In 4 cases, postoperative imaging revealed LN-clips that were placed preoperatively prior to systemic therapy indicating non-removed lymph node metastases. The treatment decisions were discussed interdisciplinary with explicit consideration of another individual surgical intervention before consensus of radiotherapy.

### Radiotherapy

All patients were treated at the Department of Radiation Oncology of the Technical University in Munich (TUM) between 01/2010 and 12/2020.

Every patient underwent planning computer tomography of the thorax (Somatom Emotion 16 scanner (Siemens Healthineers, Erlangen, Germany). All treatment plans were created in Eclipse 15.6 (Varian Medical Systems, Palo Alto, CA, USA) using VMAT technique. Contouring and treatment planning was performed according to current guidelines (18–20) and all treatment plans were approved in house by a board of attending radiation oncologists. The prescribed dose to the breast or chest wall was 50.4 Gy in 28 fractions except for two cases (51 Gy in 30 fractions/54 Gy in 30 fractions). One patient terminated radiotherapy early after a total dose of 45 Gy to the breast (25 of 28 planned fractions). N=18 patients had in addition to the LN-SIB a simultaneous integrated boost to the primary tumor region with a median dose of 59.4 Gy (range 56-64 Gy/2-2.25 Gy). Indications for an integrated boost to the tumor bed after lumpectomy were premenopausal status, ≥T2 tumor, G3 grading, HER2 positivity or triple negative breast cancers (21).

All patients received elective lymph node irradiation in addition to breast or chest wall radiotherapy. Elective lymph node irradiation comprised the supra-/infraclavicular region (level III-IV) and the internal mammary region. In addition, the axillary levels I and II were included in case of a positive SLNB/TAD or if remaining suspect lymph nodes were detected in these levels after ALND. The cervical lymph nodes were only included in case of cervical lymph node involvement. The LN-SIB-PTV was confined to radiographically suspect lymph nodes defined as GTVn plus an isotropic margin of 0.6-0.8 cm with exclusion of any lung tissue and the skin (3-5 mm from body contour). The boost dose to the LN-SIB-PTV was determined individually for each patient depending on the location and the size of the LN-SIB. The dose in the LN-SIB-PTV was prescribed to the D50% and D98% was aimed to be >95 %. The brachial plexus was contoured in every case and the dose within the plexus PRV (+0.3-0.5mm) was limited to an EQD2 of 59 Gy.

### Follow-up and data collection

All patients received structured radiooncological and gynecological follow-up by clinically experienced physicians. Intervals for radiooncological procedures follow-up were at 0 weeks, 6 weeks, 3 months, 6 months and subsequent annually after irradiation according to institutional standard operation. Gynecological follow-up was performed according to national guidelines. Every appointment at the department of radiation oncology included a full anamnesis and physical examination of the breast. Acute and late side effects were classified according to international Common Toxicity Criteria of National Cancer Institute (version 5.0). Retrospective assessment of acute side effects (radiodermatitis/desquamation, edema of the breast/arm respectively and fatigue) was performed based on the documented side effects (CTCAE) during treatment and follow-up within <6 weeks after irradiation. For evaluation of late side effects (radiodermatitis/desquamation, edema of the breast/arm, fibrosis, fatigue, brachial plexopathy, arm or shoulder pain, Pneumonitis, cardiac disease) and oncological outcome, we analyzed follow-up data ≥ 6 weeks after irradiation. For both acute and late toxicity we only report the highest toxicity grade during treatment and/or follow-up. In case of a lymph node recurrence, we evaluated spatial correlation of recurrence and LN-SIB volume.

## Results

Median follow-up time was 557 days (82.4 weeks) and ranged from 41 to 3373 days.

### LN-SIB

The median dose to the LN-SIB-PTV was 58.8 Gy/2.1 Gy (range 56-63 Gy/2-2.25 Gy). The median SIB-volume was 49.8 cm^2^ (range 5.0-759.4 cm^2^). The median number of included sLN in the SIB Volume was 5.

**Figure 1:**
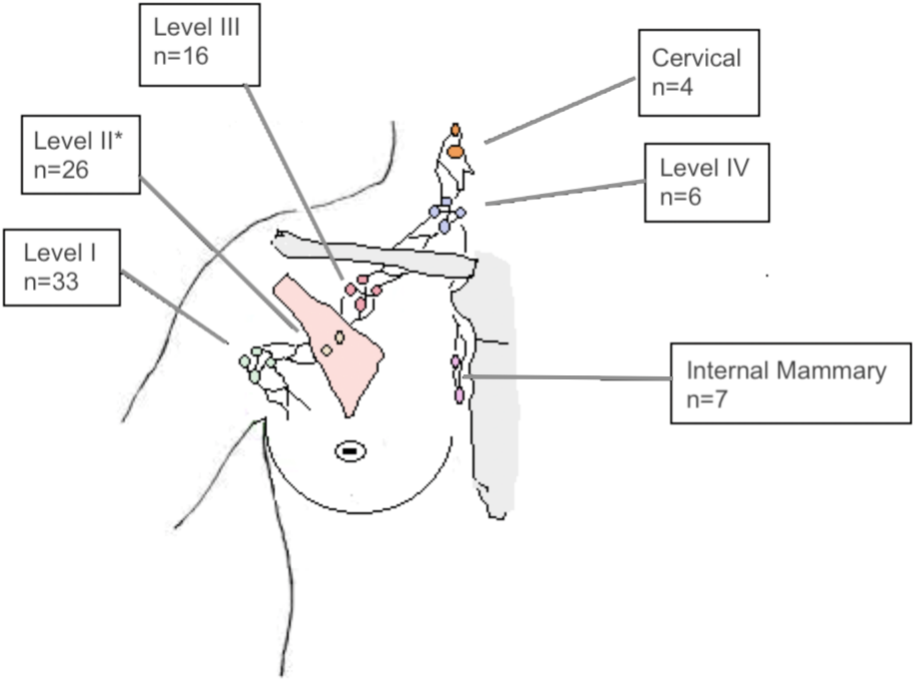
Number of patients (n=48) with LN-SIB in the axillary levels I, II, III, IV, the internal mammary region or the cervical lymph nodes. Note that LN-SIB included more than one lymph node level in 26 patients, which accounts for differences from total number of patients. *also comprises interpectoral lymph nodes.

In most patients, SIB of the sLN included only one axillary level (n=22). In 7 Patients the SIB overlapped two lymph node levels. In 7 patients three or more levels were at least partly included in the SIB volume. The localization of LN-SIB is delineated in **Figure 1**.

### Acute side effects

**Figure 2:**
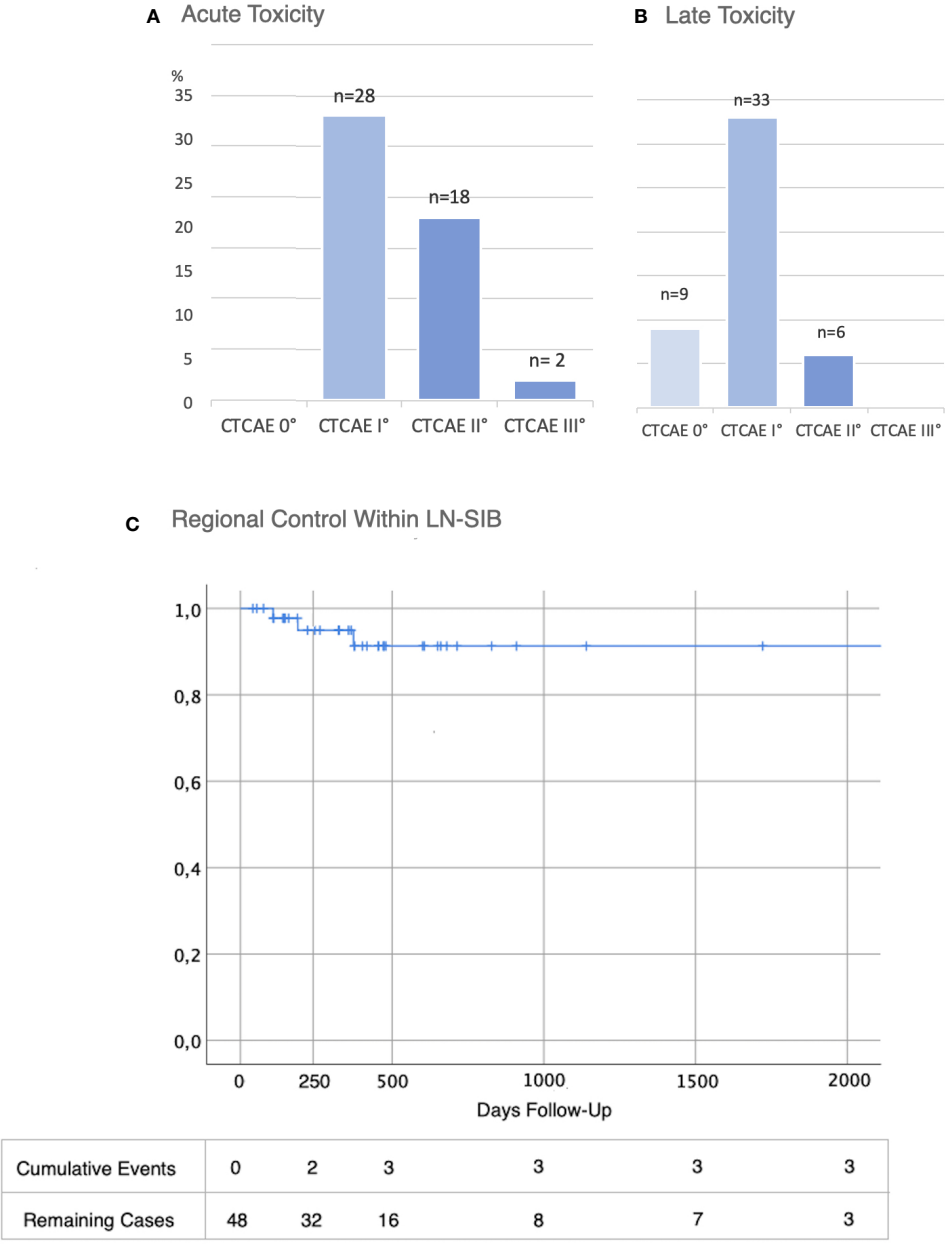
Toxicity and Oncological Outcome after LN-SIB. **(A)** Acute and **(B)** Late toxicity (CTCAE in % and total numbers (n=48)) **(C)** Regional control within LN-SIB.

Overall, 28 patients developed only I°, 18 patients II° and 2 patients III° acute toxicity. Skin reactions were observed in all patients (n=48; 100%). 95.8% (n=46) experienced only mild or moderate skin toxicity (≤ II° CTCAE). 2 patients showed severe skin toxicity. In one of them, radiotherapy had to be terminated after a total of 45 Gy due to the severe skin reactions with moist desquamation. However, the areas with moist desquamation were limited to the breast and in no proximity to the LN-SIB. Beyond this, there was no high graded toxicity. Detailed acute toxicity data is listed in **Table 1** and summarized in **Figure 2**.

**Table 1:** Summary of acute toxicity (absolute and relative numbers) classified with CTCAE.

	CTCAE			
	0°	I°	II°	III°
Radiodermatitis*	–	n=29 (60.4 %)	n=17 (35.4 %)	n=2 (4.2 %)
Edema of the breast	n=34 (70.8 %)	n=11 (22.9 %)	n=3 (6.3 %)	–
Edema of the arm	n=41 (85.4 %)	n=5 (10.4 %)	n=2 (4.2 %)	–
Fatigue	n=18 (37.5 %)	n=27 (56.3 %)	n=3 (6.3 %)	–

*Desquamation (dry or moist) occurred in n=10 patients (20.8%).

### Late side effects

There were no severe side effects (≥ III°) observed during the follow-up period. Moderate (CTCAE grade II) late toxicity was observed in six patients. The most frequent chronic side effect was fatigue, though this was only mildly expressed (**Table 2**).

**Table 2:** Summary of late effects (absolute and relative numbers) classified by CTCAE.

	CTCAE			
	0°	I°	II°	III°
Radiodermatitis*	n=44 (91.6 %)	n=3 (6.3 %)	n=1 (2.1 %)	–
Edema of the breast	n=37 (77.1 %)	n=11 (22.9 %)	–	–
Edema of the arm	n=35 (72.9 %)	n=10 (20.8 %)	n=3 (6.3 %)	–
Fibrosis	n=34 (70.8 %)	n=10 (20.8 %)	n=4 (8.3 %)	–
Fatigue	n=28 (58.3 %)	n=20 (41.7 %)	–	–
Brachial plexopathy	n=47 (97.9 %)	–	n=1 (2.1 %)	–
Pain of arm or shoulder	n=45 (93.8 %)	n=2 (4.2 %)	n=1 (2.1 %)	–
Pneumonitis	n=48 (100 %)	–	–	–
Cardiac Disease	n=48 (100 %)	–	–	–

*Dry desquamation occurred in n=2 patients (4.2 %).

Two patients suffered from polyneuropathy I° after systemic therapy. One patient developed a plexopathy with mild paresthesia immediately after radiotherapy and neuropathic pain in the ipsilateral arm after a period of 17 months. Electromyography revealed normal, however significantly reduced values compared to contralateral side. A MRI of the brachial plexus and spine showed no signs of inflammation or tumor progression but an uncovertebral arthrosis with subsequent stenosis of the spinal canal at the level of C5/6.

### Oncologic outcome

**Figure 3:**
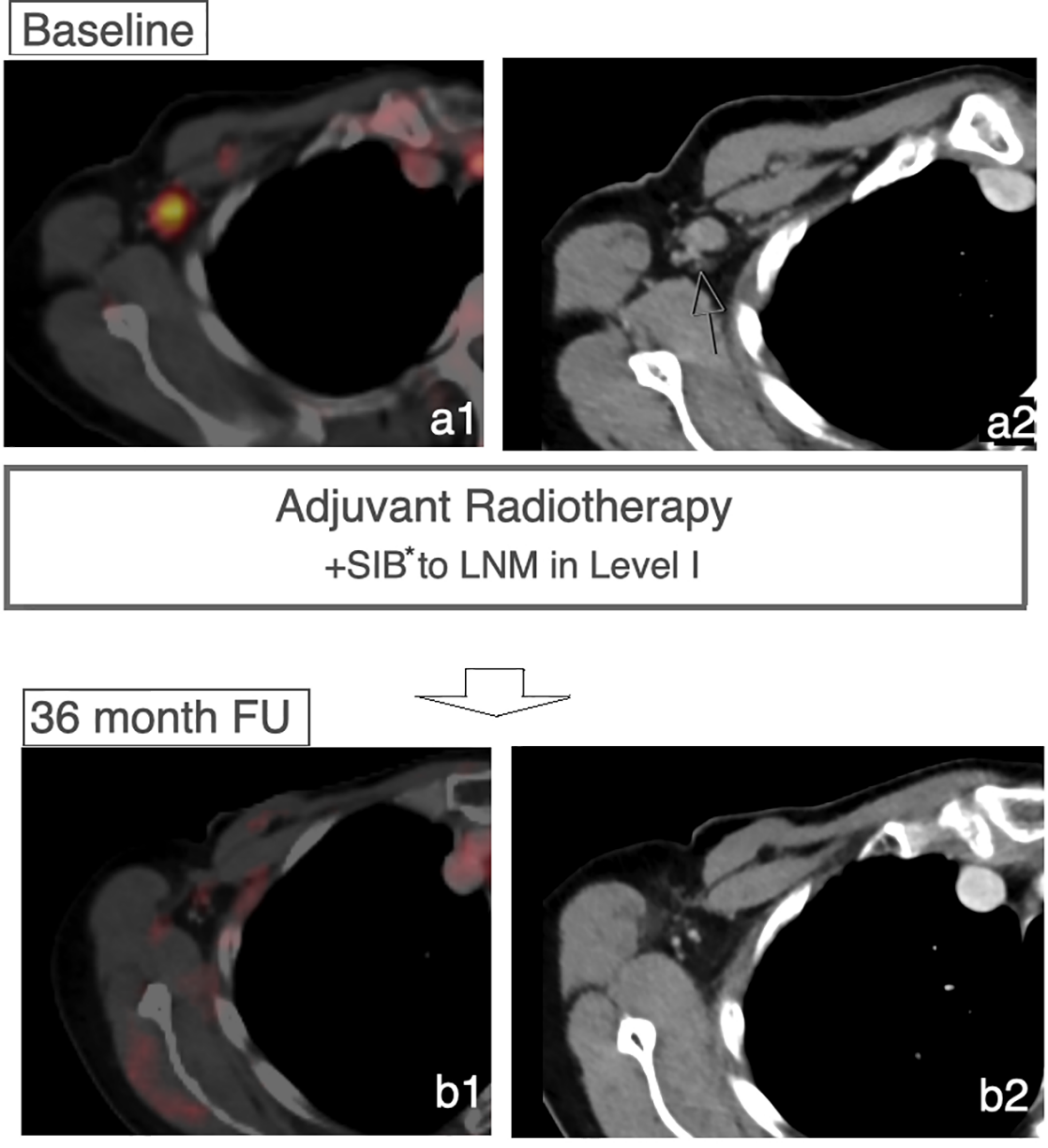
Controlled LN after LN-SIB. Case of a patient receiving a LN-SIB to a remaining sLN in Level I. FDG-PET/CT imaging after 36 months shows regression without signs of recurrence within the lymphatic drainage system. *Dose 58.8 Gy in 28 Fx.

After a median follow-up of 557 days, 40 patients had neither a breast cancer recurrence nor progress of the LN within the LN-SIB (**Figure 3**).

**Figure 4:**
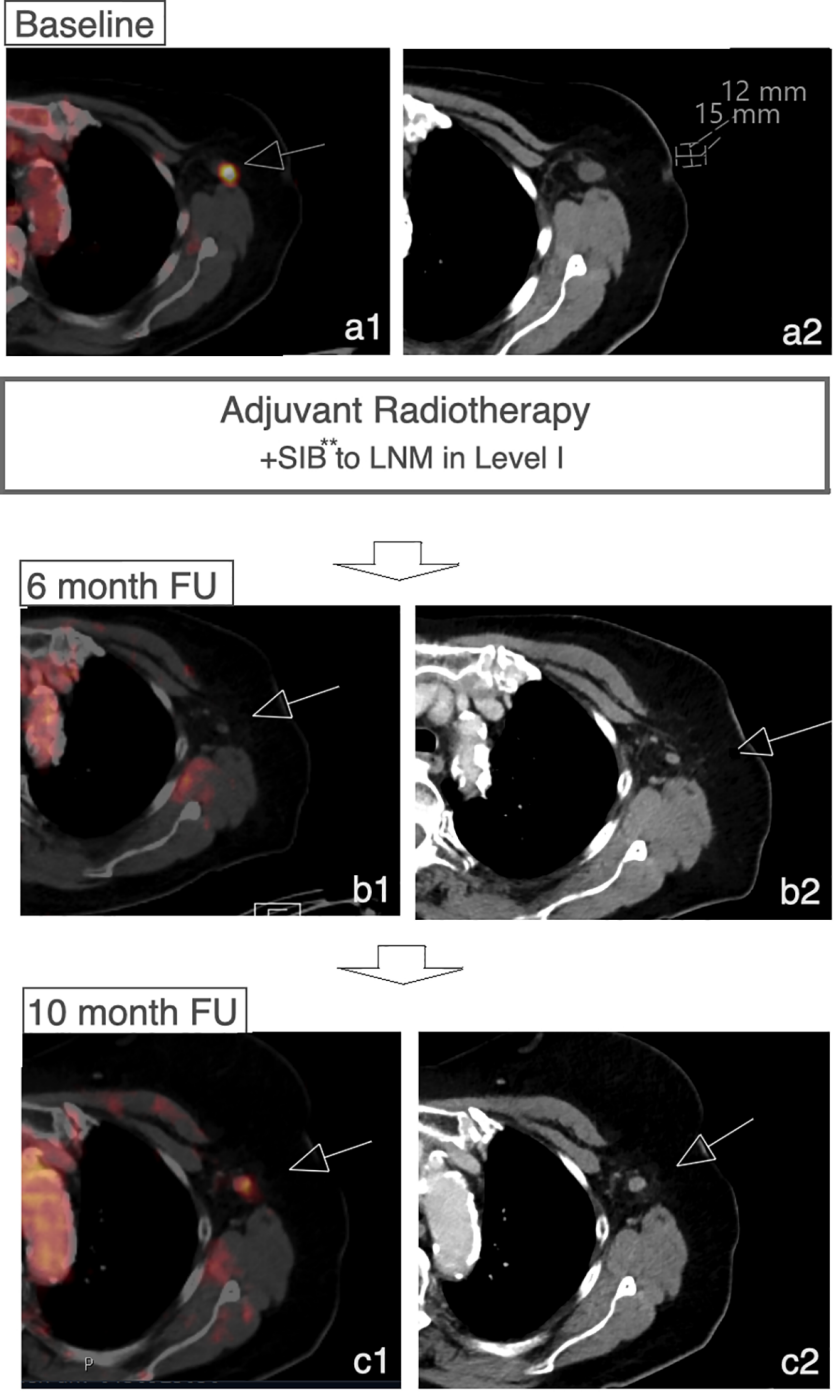
Recurrence within LN-SIB. Case of a patient with initial response to LN-SIB of a LN in level I 3 months after RTx and recurrence 10 months after RTx. **Dose 58.8 Gy in 28 Fx.

In 8 cases patients developed a recurrence during the follow-up period (16.7%). In 4 patients, recurrence involved the regional lymph node system. The remaining patients developed local recurrence in the breast (n=1) or distant metastases (n=3) without recurrence or progress within the regional lymph node system. Of 4 patients with recurrence within the regional lymph node system 3 patients developed a recurrence or progress within the LN-SIB target volume (**Figure 4**; **Figure 2C**). The median time to recurrence within the LN-SIB volume was 189 days (108 to 373 days). Patients with lymphatic recurrence within LN-SIB volume had slightly larger SIB target volumes (54.7 cm^2^ vs. 49.8 cm^2^) and a slightly lower prescription dose (median: 56.25 Gy/2.1 Gy; range 56-58.8 Gy/2-2.25 Gy) compared to the remaining patients.

## Discussion

With this study, we present preliminary data for the use of SIB-irradiation on sLN in patients with invasive breast cancer. Assessment of acute and late toxicity showed mostly mild or moderate skin reactions, fatigue and edema of the breast or arm. There were no late toxicities reported higher than II° according to CTCAE. However, there was one case of brachial plexopathy of unknown cause. Despite residual lymph node metastases prior to radiotherapy only 3 patients developed a recurrence within the LN-SIB volume after a median follow-up of > 1.5 years.

With the increasing de-escalation of axillary surgery, evidence for tailored radiotherapy approaches to the axilla is urgently needed.

The most comprehensive data regarding toxicity of lymph node irradiation exist from the randomized EORTC 22922, MA 20 trial and AMAROS trial (2, 4, 22). The toxicity rates of EORTC 22922 and MA.20 trial were comparable to the toxicity rates of our current trial:

In the EORTC 22922 trial, chronic skin toxicity was found in 13.6% of all patients after irradiation of internal mammary and medio supraclavicular lymph nodes, including fibrosis in 7.9% and radiodermatitis in 1.4%. Edema of the breast, chest wall or arm was observed in 4.2% of patients. No information was provided on acute side effects. It should be noted that in these studies only 7.4% (control group) respectively 8.3% (nodal irradiation group) of patients were underdoing axillary irradiation.

In the MA.20 trial side effects were only reported from moderate or higher intensity (≥II°). Acute skin reactions were found in 49.5% of patients; fatigue occurred in 19% of patients. Chronic skin reactions were reported in 6.9%. Lymphedema could be objected in 8.4% of patients.

For the AMAROS trial in which full dose coverage to the axillary levels I and II was given in the treatment arm, a lymphedema rate of 15 % was reported after one year. The lower rate of lymphedema compared to our trial is most likely attributed to the fact that patients in the treatment arm received “SLNB only”, whereas in our study most patients received ALND. A recent study by Naoum et al. showed that the type of axilla-surgery is the most important risk factor for the development of lymphedema and regional radiotherapy has only a minor impact (23).

One case of brachial plexopathy of unknown cause occurred during follow-up. Even though the immediate onset of paresthesia and the normal plexus-MRI are not typical for radiogenic plexopathy and the symptoms could also be related to a diagnosed uncovertebral arthrosis, a causal relationship cannot be ruled out. Meta-analyses have shown that brachial plexus constraints of EQD2 60–66 Gy are safe even when hypofractionated RT is being used (24). It should be noted that the dose distribution in the MA.20, the EORTC and the AMAROS trial was more inhomogeneous compared to modern treatment planning. In a previous study we showed that the field design of the AMAROS trial resulted in median doses up to >56 Gy in the axillary levels I and II (25). Taking this into account a LN-SIB with a dose of 58.8 Gy and brachial plexus sparing using modern 3D-based VMAT treatment planning might lead to similar maximal doses to the lymphatic drainage system and the brachial plexus compared patients included in the randomized AMAROS trial.

In earlier trials, the median time of brachial plexopathy after radiotherapy was 7 months. Therefore, the follow-up data regarding brachial plexopathy in our study (2.1 %) is relevant despite short follow-up of only 1.5 years.

Nevertheless, since long-term follow-up data is lacking, patients receiving a LN-SIB should be informed about a higher risk of brachial plexopathy due to the dose-response relationship of brachial plexopathy. This applies particularly to patients receiving large LN-SIBs as there is emerging evidence that there is a volume effect associated with the brachial plexus (26).

We observed recurrence of the primary tumor region in 4.2%. Lymphatic recurrences were diagnosed in 8.3 %. In 6.25% recurrence occurred within the LN-SIB. In comparison, the MA-20 trial reported locoregional recurrence in 4.3%. In EORTC trial, a local recurrence was found in 5.6%, a regional recurrence in 2.7%. Recurrence rate in AMAROS trial was 1.2 % in axillary radiotherapy group after 5 years. However, the follow-up in these studies was much longer (AMAROS trial: 6.1 years, MA20 trial: median 9.5 years, EORTC trial: 10 years). Thus, recurrence data from our study exceeded preliminary data from mentioned trials. The differences can be explained by an unfavorable patient collective with inclusion of recurrent and even metastatic patient*s*. Further residual macroscopic lymph node metastases after surgery are a risk factor per se. Given the high-risk features of our patient collective, a local control within the LN-SIB volume of 94 % after >1.5 years median follow-up seems reasonable. However, it can be assumed that higher doses are necessary to achieve persisting long-term tumor control in the lymph node system. Previous studies focusing on incomplete or unresected breast cancer showed that doses of EQD2 >76 Gy are necessary to achieve control rates that are comparable to control rates of surgery (27, 28). Since the prescribed dose to remaining lymph nodes in the axilla is limited by the tolerance of the brachial plexus and the risk of lymphopathy, surgery of macroscopically remaining lymph nodes is the undisputed gold standard. However, the current study shows that LN-SIB represents a potential alternative treatment option if LN-dissection or picking cannot be carried out or is refused by the patient. The limitations of the current studies, apart from the retrospective nature, are the short follow-up, the insufficient correlation of the toxicity with follow-up time and SIB location and an incomplete recording of some side effects (e.g. telangiectasias, hyperpigmentation). To draw definitive conclusion on this topic a longer follow-up and prospective randomized trials are needed.

## Conclusion

Surgical re-dissection is the undisputed gold standard for residual lymph node metastases after breast cancer surgery. If re-dissection of residual lymph nodes is not feasible or refused by the patient, LN-SIB-irradiation can be considered as a potential treatment option. However, patients need to be informed about a higher risk of regional recurrence compared to surgery and an additional risk of acute and late toxicity compared to adjuvant radiotherapy without regional dose escalation.

## Data availability statement

The raw data supporting the conclusions of this article will be made available by the authors, without undue reservation.

## Ethics statement

The studies involving human participants were reviewed and approved by Ethik-Kommission der Fakultät für Medizin der Technischen Universität München. Written informed consent for participation was not required for this study in accordance with the national legislation and the institutional requirements.

## Author contributions

KB and AP initiated the project. AP, SK and KB were responsible for data generation and analysis. SK and KB wrote the manuscript. GS, MK, SM, RA and SC advised and corrected the manuscript. All authors read and approved the final manuscript. All authors contributed to the article and approved the submitted version.

## References

[1] Carter CL Allen C Henson DE. Relation of tumor size, lymph node status, and survival in 24,740 breast cancer cases. Cancer (1989) 63(1):181–7. doi: 10.1002/1097-0142(19890101)63:1<181::AID-CNCR2820630129>3.0.CO;2-H 2910416

[2] Whelan TJ Olivotto IA Parulekar WR Ackerman I Chua BH Nabid A. Regional nodal irradiation in early-stage breast cancer. N Engl J Med (2015) 373(4):307–16. doi: 10.1056/NEJMoa1415340 PMC4556358 26200977

[3] Budach W Bölke E Kammers K Gerber PA Nestle-Krämling C Matuschek C. Adjuvant radiation therapy of regional lymph nodes in breast cancer - a meta-analysis of randomized trials- an update. Radiat Oncol (2015) 10:258. doi: 10.1186/s13014-015-0568-4 26691175 PMC4687086

[4] Donker M van Tienhoven G Straver ME Meijnen P van de Velde CJH Mansel RE. Radiotherapy or surgery of the axilla after a positive sentinel node in breast cancer (EORTC 10981-22023 AMAROS): A randomised, multicentre, open-label, phase 3 non-inferiority trial. Lancet Oncol (2014) 15(12):1303–10. doi: 10.1016/S1470-2045(14)70460-7 PMC4291166 25439688

[5] Giuliano AE Ballman KV McCall L Beitsch PD Brennan MB Kelemen PR. Effect of axillary dissection vs no axillary dissection on 10-year overall survival among women with invasive breast cancer and sentinel node metastasis: The ACOSOG Z0011 (Alliance) randomized clinical trial. JAMA (2017) 318(10):918–26. doi: 10.1001/jama.2017.11470 PMC5672806 28898379

[6] Jagsi R Chadha M Moni J Ballman K Laurie F Buchholz TA. Radiation field design in the ACOSOG Z0011 (Alliance) trial. J Clin Oncol (2014) 32(32):3600–6. doi: 10.1200/JCO.2014.56.5838 PMC4220042 25135994

[7] Veronesi U Paganelli G Viale G Luini A Zurrida S Galimberti V. A randomized comparison of sentinel-node biopsy with routine axillary dissection in breast cancer. N Engl J Med (2003) 349(6):546–53. doi: 10.1056/NEJMoa012782 12904519

[8] Ebner F Wöckel A Schwentner L Blettner M Janni W Kreienberg R. Does the number of removed axillary lymphnodes in high risk breast cancer patients influence the survival? BMC Cancer (2019) 19(1):90. doi: 10.1186/s12885-019-5292-2 30658597 PMC6339270

[9] Brackstone M Baldassarre FG Perera FE Cil T Chavez Mac Gregor M Dayes IS. Management of the axilla in early-stage breast cancer: Ontario health (Cancer care Ontario) and ASCO guideline. J Clin Oncol (2021) 39(27):3056–82. doi: 10.1200/JCO.21.00934 34279999

[10] Caudle AS Yang WT Krishnamurthy S Mittendorf EA Black DM Gilcrease MZ. Improved axillary evaluation following neoadjuvant therapy for patients with node-positive breast cancer using selective evaluation of clipped nodes: Implementation of targeted axillary dissection. J Clin Oncol (2016) 34(10):1072–8. doi: 10.1200/JCO.2015.64.0094 PMC4933133 26811528

[11] Kuemmel S Heil J Rueland A Seiberling C Harrach H Schindowski D. A prospective, multicenter registry study to evaluate the clinical feasibility of targeted axillary dissection (TAD) in node-positive breast cancer patients. Ann Surg (2020) 276(5):e553–62. doi: 10.1097/SLA.0000000000004572 33156057

[12] Banys-Paluchowski M Gasparri ML de Boniface J Gentilini O Stickeler E Hartmann S. Surgical management of the axilla in clinically node-positive breast cancer patients converting to clinical node negativity through neoadjuvant chemotherapy: Current status, knowledge gaps, and rationale for the EUBREAST-03 AXSANA study. Cancers (Basel) (2021) 13(7):1565. doi: 10.3390/cancers13071565 33805367 PMC8037995

[13] Gradishar WJ Moran MS Abraham J Aft R Agnese D Allison KH. NCCN guidelines® insights: Breast cancer, version 4.2021. J Natl Compr Canc Netw (2021) 19(5):484–93. doi: 10.6004/jnccn.2021.0023 34794122

[14] Hörner-Rieber J Forster T Hommertgen A Haefner MF Arians N König L. Intensity modulated radiation therapy (IMRT) with simultaneously integrated boost shortens treatment time and is noninferior to conventional radiation therapy followed by sequential boost in adjuvant breast cancer treatment: Results of a Large randomized phase III trial (IMRT-MC2 trial). Int J Radiat Oncol Biol Phys (2021) 109(5):1311–24. doi: 10.1016/j.ijrobp.2020.12.005 33321192

[15] Caudell JJ Gillison ML Maghami E Spencer S Pfister DG Adkins D. NCCN guidelines® insights: Head and neck cancers, version 1.2022. J Natl Compr Canc Netw (2022) 20(3):224–34. doi: 10.6004/jnccn.2022.0016 35276673

[16] Schaeffer E Srinivas S Antonarakis ES Armstrong AJ Bekelman JE Cheng H. NCCN guidelines insights: Prostate cancer, version 1.2021. J Natl Compr Canc Netw (2021) 19(2):134–43. doi: 10.6004/jnccn.2021.0008 33545689

[17] Abu-Rustum NR Yashar CM Bean S Bradley K Campos SM Chon HS. NCCN guidelines insights: Cervical cancer, version 1.2020. J Natl Compr Canc Netw (2020) 18(6):660–6. doi: 10.6004/jnccn.2020.0027 32502976

[18] Offersen BV Boersma LJ Kirkove C Hol S Aznar MC Sola AB. ESTRO consensus guideline on target volume delineation for elective radiation therapy of early stage breast cancer. Radiother Oncol (2015) 114(1):3–10. doi: 10.1016/j.radonc.2014.11.030 25630428

[19] White J Tai A Arthur D Buchholz T MacDonald S Marks L. RTOG. breast cancer atlas for radiation therapy planning. Philadelphia, Pennsylvania, USA: RTOG (2008). Available at: https://www.rtog.org/LinkClick.aspx?fileticket=vzJFhPaBipE%3d&tabid=236.

[20] WJ G. NCCN guidelines breast cancer (Version 3.2022) (2022). Available at: https://www.nccn.org/professionals/physician_gls/pdf/breast.pdf.

[21] Leitlinienprogramm onkologie | S3-leitlinie mammakarzinom| version 4.3 (2021). Available at: https://www.leitlinienprogramm-onkologie.de/fileadmin/user_upload/Downloads/Leitlinien/Mammakarzinom_4_0/Version_4.4/LL_Mammakarzinom_Kurzversion_4.3.pdf.

[22] Poortmans PM Collette S Kirkove C Van Limbergen E Budach V Struikmans H. Internal mammary and medial supraclavicular irradiation in breast cancer. N Engl J Med (2015) 373(4):317–27. doi: 10.1056/NEJMoa1415369 26200978

[23] Naoum GE Roberts S Brunelle CL Shui AM Salama L Daniell K. Quantifying the impact of axillary surgery and nodal irradiation on breast cancer-related lymphedema and local tumor control: Long-term results from a prospective screening trial. J Clin Oncol (2020) 38(29):3430–8. doi: 10.1200/JCO.20.00459 PMC7527159 32730184

[24] Yan M Kong W Kerr A Brundage M. The radiation dose tolerance of the brachial plexus: A systematic review and meta-analysis. Clin Transl Radiat Oncol (2019) 18:23–31. doi: 10.1016/j.ctro.2019.06.006 31309161 PMC6606964

[25] Borm KJ Oechsner M Düsberg M Buschner G Weber W Combs SE. Irradiation of regional lymph node areas in breast cancer - dose evaluation according to the Z0011, AMAROS, EORTC 10981-22023 and MA-20 field design. Radiother Oncol (2020) 142:195–201. doi: 10.1016/j.radonc.2019.08.021 31540747

[26] Lundstedt D Gustafsson M Steineck G Sundberg A Wilderäng U Holmberg E. Radiation therapy to the plexus brachialis in breast cancer patients: Analysis of paresthesia in relation to dose and volume. Int J Radiat Oncol Biol Phys (2015) 92(2):277–83. doi: 10.1016/j.ijrobp.2015.01.016 25765147

[27] Borger JH van Tienhoven G Passchier DH Hart AA van Dongen JA Rutgers EJ. Primary radiotherapy of breast cancer: Treatment results in locally advanced breast cancer and in operable patients selected by positive axillary apex biopsy. Radiother Oncol (1992) 25(1):1–11. doi: 10.1016/0167-8140(92)90188-Z 1410583

[28] Shibamoto Y Murai T Suzuki K Hashizume C Ohta K Yamada Y. Definitive radiotherapy with SBRT or IMRT boost for breast cancer: Excellent local control and cosmetic outcome. Technol Cancer Res Treat (2018) 17. doi: 10.1177/1533033818799355 PMC6141921 30222523

